# Walking Behavior of Zoo Elephants: Associations between GPS-Measured Daily Walking Distances and Environmental Factors, Social Factors, and Welfare Indicators

**DOI:** 10.1371/journal.pone.0150331

**Published:** 2016-07-14

**Authors:** Matthew R. Holdgate, Cheryl L. Meehan, Jennifer N. Hogan, Lance J. Miller, Joseph Soltis, Jeff Andrews, David J. Shepherdson

**Affiliations:** 1 Department of Biology, Portland State University, Portland, Oregon, United States of America; 2 Conservation Research Division, Oregon Zoo, Portland, Oregon, United States of America; 3 AWARE Institute, Portland, Oregon, United States of America; 4 Chicago Zoological Society—Brookfield Zoo, Brookfield, Illinois, United States of America; 5 Department of Education & Science, Disney’s Animal Kingdom, Lake Buena Vista, Florida, United States of America; 6 Zoological Operations, Busch Gardens, Tampa, Florida, United States of America; University of Florida, UNITED STATES

## Abstract

Research with humans and other animals suggests that walking benefits physical health. Perhaps because these links have been demonstrated in other species, it has been suggested that walking is important to elephant welfare, and that zoo elephant exhibits should be designed to allow for more walking. Our study is the first to address this suggestion empirically by measuring the mean daily walking distance of elephants in North American zoos, determining the factors that are associated with variations in walking distance, and testing for associations between walking and welfare indicators. We used anklets equipped with GPS data loggers to measure outdoor daily walking distance in 56 adult female African (n = 33) and Asian (n = 23) elephants housed in 30 North American zoos. We collected 259 days of data and determined associations between distance walked and social, housing, management, and demographic factors. Elephants walked an average of 5.3 km/day with no significant difference between species. In our multivariable model, more diverse feeding regimens were correlated with increased walking, and elephants who were fed on a temporally unpredictable feeding schedule walked 1.29 km/day more than elephants fed on a predictable schedule. Distance walked was also positively correlated with an increase in the number of social groupings and negatively correlated with age. We found a small but significant negative correlation between distance walked and nighttime Space Experience, but no other associations between walking distances and exhibit size were found. Finally, distance walked was not related to health or behavioral outcomes including foot health, joint health, body condition, and the performance of stereotypic behavior, suggesting that more research is necessary to determine explicitly how differences in walking may impact elephant welfare.

## Introduction

The distance that zoo elephants walk each day has been proposed as a biologically meaningful metric for measuring the success of zoo elephant programs in providing good welfare for their elephants [1]. Implicit in this recommendation is an assumption that elephants are strongly motivated and physiologically adapted to walk long distances, and that the welfare of zoo elephants is therefore compromised when walking distance is constrained, for example due to the size of the exhibit. The walking behavior of wild elephants has been measured under various conditions using a variety of techniques (summarized in [2]). However, there is significant variation in measured distances both within and between these studies, with reported daily walking distances ranging from 3.2 to 12.0 km/day [2]. Variation in wild elephant movement is affected by a variety of factors including the age and sex of the individual, season, social groupings, and the distribution and availability of resources [3–5]. In some cases these relationships encourage greater amounts of walking; elephants can travel long distances to seek out fruiting events [6], sodium [7–8], and green vegetation [9]. However, when conditions dictate, elephants’ locomotion is more limited. In the dry season, African elephant family units are unable to range far from water, and bulls in musth remain close to family units to maximize mating opportunities [10]. The link between resource availability and walking distances suggests that walking is an effective means for meeting social or nutritional needs and is performed flexibly in response to external conditions. In zoo settings, social and nutritional needs are addressed through management practices and therefore, the functional need for walking in this context is reduced. However, it is also possible that walking is important because it supports exploratory behavior which has an information-gathering function and may be rewarding to animals even when not directly linked to acquisition of resources [11–12]. Rats, for example, have been found to forgo sucrose rewards in order to physically investigate their environment [13], but studies of elephants’ motivation to explore via locomotion have not been conducted.

In order to better understand the walking behavior of zoo elephants and its importance to welfare, it is necessary to develop methods to reliably collect data on the distances elephants walk under a range of conditions, and to determine the demographic, social, environmental, and management-related factors that are associated with differences in distances walked. Finally, it is important to test for associations between distances walked and other behavioral or health indicators to validate walking as a measure of welfare [14].

Physical exercise (including, but not limited to walking) is known to be associated with good health in humans (e.g., [15–18]), and this relationship has also been measured in other species. For example, dogs that experienced vigorous exercise sessions had fewer behavioral and physiological signs of stress than their unexercised counterparts [19]. With respect to elephants, a survey-based study of 78 zoos found that zoos providing their elephants with more daily exercise reported fewer incidents of foot pathology [20]; another study [21] found that zoo elephants who experienced 14 hours per week of exercise had a lower likelihood of being overweight or obese. The challenge in applying the results of these studies to the relationship between walking and zoo elephant welfare is that they evaluated human-led exercise, which could have different associations with welfare than self-directed exercise. Human-led exercise sessions were associated with an increase in the performance of stereotypic behavior in horses [22]. In preference tests, horses reliably chose to remain in their stalls rather than participate in human-led exercise [23], but also preferred voluntary exercise (via release into a large paddock) over remaining in their stall [23]. The health outcomes of voluntary walking have been studied in cattle, and foot and musculoskeletal problems, mastitis, and dystocia were found to be significantly lower for cows that were given access to pasture to areas large enough to allow free-choice exercise [24]. There are no published studies investigating the relationships between walking distances and the welfare of zoo elephants.

The objectives for the current study were to: 1) use GPS tracking to quantify and describe the outdoor daily walking distances for both African and Asian elephants from a variety of zoos, 2) determine the associations between housing, social, management, and demographic factors and distance walked, and 3) test for associations between distance walked and a variety of health and behavioral welfare indicators including: foot health and musculoskeletal health, performance of stereotypic behavior, and body condition. Our study is the first large-scale multi-species investigation of zoo elephant walking and was as a component of the *Using Science to Understand Zoo Elephant Welfare* project, a multi-institutional collaborative effort to produce scientific data that will support decision making with regard to best practices in elephant management [25].

## Methods

### Ethics statement

This study was authorized by the management at each participating zoo and, where applicable, was reviewed and approved by zoo research committees. In addition, the study protocol was reviewed and approved by the Zoological Society of San Diego Institutional Animal Care and Use Committee N.I.H. Assurance A3675-01; Protocol 11–203. The study was non-invasive.

### Subjects and facilities

Zoos that were accredited members of the Association of Zoos and Aquariums in 2012 were eligible for participation in this study provided that they managed African or Asian elephants in a non-mixed species herd, and that their herd included at least two adult female elephants who were not pregnant or experiencing severe illness or injury. A total of 49 zoos participated in the study. We used simplified random sampling to select two adult females (age ≥ 12 years) as subjects from each zoo; however, 26 zoos only had two eligible subjects so there was no randomization. In one case there were four subjects from one zoo because this zoo housed African and Asian elephants in separate exhibits. Three subjects were removed from the dataset prior to analysis because they were transferred between zoos or died during the 2012 study year.

### Data collection

All data were collected between May 2012 and November 2012. We used historical weather data [26] to select a one month data collection period at each location that minimized inter-zoo variation in predicted daily maximum temperature (range: 22.3 C to 34.1 C). We instructed zoos to collect five non-consecutive days of data (24 hours/day) from each subject within that one-month timeframe. Zoos could collect data from both subjects on the same day, or use an alternating schedule. Zoos also completed detailed housing logs on the days of data collection indicating the areas to which elephants had access to and whether these areas were indoors, outdoors, or a mixed (indoor-outdoor) environment.

Leather anklets (Excelsior Leather, California, USA) [27–28] were custom-fit to elephants using measurements provided by participating zoos. The ends of the anklets had D-rings to which shackles and brummel hooks were attached. This hardware was used to secure the anklet in place without causing constriction. A pouch attached to each anklet contained a waterproof case (OtterBox Drybox OTR3-1000S, OtterBox, Colorado, USA) inside of which was an accelerometer (used to collect data for a related study [29]) and a BT-Q1000XT GPS Travel Recorder (QStarz International Co., Taipei, Taiwan) (for anklet photo see [28]). The BT-Q1000XT has been used in previous zoo elephant research [28] and tests indicate that this device can be used to accurately assess elephant movement in a zoo environment [28]. The total weight of the unit was approximately 1.2 kg depending on the anklet size and number of shackles used. We shipped the anklets to the zoos and elephant care staff attached the anklets to one of the front legs of each subject. Most studies of zoo elephant movement have used collars to attach GPS devices [3,27,30–32]; anklets were preferred for our multi-institutional study because they require less time for elephants to habituate to them and they are safer for zoo staff to place on the elephants.

We programmed the GPS units to record data points at five second intervals in accordance with a previous study using these units [28]. Each data point includes the date, time, latitude, longitude, and two indices of location estimate quality: the number of satellites used (NSAT) and the geometry of satellites used (HDOP: horizontal dilution of precision; [33]) in each location estimate. The device also received real-time positional corrections to improve accuracy via wide area augmentation system (WAAS; [34]). Finally, we requested that zoos place anklets in an exposed outdoor area fifteen minutes prior to data collection to provide sufficient time for the device to download satellite constellation information.

### Data processing

Of the 49 original participating zoos, 40 zoos successfully collected data from 72 elephants. We downloaded the data using QTravel software (v 1.41, QStarz International Co.) and exported it into Microsoft Excel (Microsoft Corporation, Washington, USA). Since the GPS units do not reliably collect data when indoors, we removed data points that were known to have occurred while the elephant was housed indoors by using detailed reports on indoor/outdoor access provided by the zoos. We then mapped the data using ArcMap (v. 10.1, Environmental Systems Research Institute, California, USA) and used the clip function to remove any remaining data from indoor areas. We also clipped any data that fell outside of exhibit boundaries; in one test case we found that these data had a minimal effect (3%) on distance traveled, but clipping the data ensured that we underestimated rather than overestimated the distance. We then filtered the data using a series of macros. Data points were removed if they failed to meet any of the following requirements: NSAT ≥4, HDOP <2, WAAS-enabled.

Following this processing, the amount of valid, outdoor walking data collected from the elephants in our study varied greatly. Thus, we applied additional exclusion criteria to ensure that all elephants proceeding to the analysis stage met a minimum standard. First, in order for their data to be included in the analysis elephants were required to have outdoor access for at least 20/24 hours; any 24 hour period that did not meet this criterion was excluded. This helped minimize the potential effect of long bouts of confinement indoors, since post-inhibitory “rebound” effects can cause increased locomotor activity following confinement in some species [35]. Second, elephants were required to have a total of at least 60 minutes of valid data for each one-hour period across all days of data collection (except for the 0–4 hours they did not have outdoor access). This allowed us to remove any bias due to elephants who had consistently missing data from the same time periods each day. Finally, elephants with less than 3 days of data remaining after quality control filtering were excluded from analysis. A total of 16 elephants were removed during data processing; our final dataset included 56 elephants from 30 zoos. For these elephants we calculated the Euclidean distance between consecutive data points and we summed the distances for each day to calculate a daily distance traveled (km). Finally, we averaged these daily values to calculate mean daily walking distance (MDWD) (km/day) for each elephant.

### Independent variables

Independent variables were selected based on hypotheses regarding their potential association with MDWD. Definitions for the variables selected for testing in this study are described in Table 1. Details on the collection and calculation of independent variables are presented by [29, 36–38].

**Table 1:** Definitions of independent variables tested for correlation with mean daily walking distance.

Variable	Unit of Analysis	Unit	Time Scale	Description	Ref
Age	Elephant			Age of elephant (years)	[38]
Species	Elephant			African savanna or Asian	[38]
Origin	Elephant			Captive or wild born	[38]
Total Exhibit Size	Zoo	500 ft^2^		Total area of space available to elephants at zoo	[36]
Space Experience				The average weighted (by percent time) size of all environments in which an elephant spent time	[36]
Total	Elephant	500 ft^2^	Overall, Day, Night	For all environment types	[36]
Indoor	Elephant	500 ft^2^	Overall, Day, Night	For indoor environments only	[36]
In/Out Choice	Elephant	500 ft^2^	Overall, Day, Night	For environments where there is a choice of indoors or outdoors	[36]
Outdoor	Elephant	500 ft^2^	Overall, Day, Night	For outdoor environments only	[36]
Percent Time				Sum of monthly percent time spent in category, averaged over time period	
Soft Substrate	Elephant	%	Overall, Day, Night	Time spent in environment with 100% grass, sand, or rubber substrate	[36]
Hard Substrate	Elephant	%	Overall, Day, Night	Time spent in environment with 100% concrete or stone aggregate substrate	[36]
Housed Separately	Elephant	%	Overall, Day, Night	Time spent housed in a social group of one	[36]
Juveniles (<7 years old)	Elephant	%	Overall, Day, Night	Time spent in social groups where an elephant 7 years or younger was present	[36]
Social Experience	Elephant		Overall, Day, Night	The average weighted (by percent time) size of all social groups in which an elephant spent time	[36]
Animal Contact	Elephant		Overall, Day, Night	Max number of unique elephants focal animal is in contact with	[36]
Social Group Contact	Elephant			Max number of unique social groups focal animal is part of	[36]
Recumbence	Elephant			Hours recumbent per day, averaged over all days of data collection	[29]
Herd Size	Zoo			Total number of elephants at zoo	[36]
Temperature	Zoo			Average daily temperature at zoo, averaged over all days of data collection	
Enrichment Program	Zoo			Standardized factor score created using a polychoric PCA to examine the frequency of use of the different components of an enrichment program	[37]
Enrichment Diversity	Zoo			Shannon diversity index score of enrichment activities types and frequencies conducted at zoo	[37]
Exercise Diversity	Zoo			Shannon diversity index score of exercise types and frequencies conducted at zoo	[37]
Feeding Diversity	Zoo			Shannon diversity index score of feeding types and frequencies conducted at zoo	[37]
Feeding Predictability	Zoo			The predictability of feeding times; categorical where 1 is predictable, 2 is semi-predictable, and 3 is unpredictable	[37]
Feed Total	Zoo			Sum of the number of feedings during the day and number of feedings during the night	[37]
Alternate Feeding Types	Zoo			The proportion of all feedings where food was presented in a foraging device, hidden, or hung above the exhibit	[37]
Spread	Zoo			The proportion of all feedings where food was spread through the exhibit	[37]

A novel variable called Space Experience warrants further attention. Space Experience was calculated by taking the size of each environment in which an elephant spent time, multiplying it by the percentage of time the elephant spent in that environment and then averaging these weighted environment sizes [36]. This allows us to account for the complex housing conditions of zoo elephants, in which they may be shifted between environments of different sizes for varying amounts of time, including at night.

We checked all continuous independent variables for outliers and removed any values that were greater than three standard deviations away from the mean. We adjusted some variables from continuous to binary because of zero-values for a high number of subjects within the sub-population of elephants in our study. Adjusted variables included two space variables (Space Experience In/Out Choice and Percent Time In/Out Choice), two flooring variables (Percent Time Hard Substrate and Percent Time Soft Substrate) and two social variables (Percent Time Housed Separately and Percent Time Juveniles). The Space Experience variables were adjusted to a value of “per 500 ft^2^” to aid in the interpretation of Beta values.

### Statistical analysis

The regression models were fitted using generalized estimating equations (GEE), which allow for the individual elephant to be used as the unit of analysis, account for clustering of individuals within zoos, and support repeated measurement [39–40]. Zoos were treated as random effects and an independent correlation structure was specified [41]. We first built multi-variable regression models by assessing individual predictors at the univariate level and then at the bivariate level with demographic variables (age, species and origin). Based on the fact that age, species and origin were likely to have an effect on both outcome and the tested input variable, we tested these variables as potential confounding variables [42–43]. Confounding variables (those that altered the beta values of input variables by more than 10% during bivariate analysis) were included in all models, and any variables that predicted recumbence (P < 0.15) following the univariate and bivariate assessments were retained for evaluation in the hierarchical model building process. The hierarchical selection was based on quasi-likelihood under the independence model criterion (QIC) values and parameter estimates of explanatory variables.

Models exhibiting multi-collinearity, as defined by a variance inflation factor (VIF) of greater than 10 and a Condition Index (CI) of greater than 30, were not considered for further analysis. The model used an independent correlation matrix type. Statistical analyses were conducted by using SAS software, version 9.3 [PROC GENMOD, with options REPEATED, CORR = IND, and DIST = NORMAL; SAS Institute, Inc., Cary, NC].

Regression analyses were used to assess the association between MDWD as an input parameter and other outcomes measured in associated studies. These are Foot Health Score [44], Musculoskeletal Health Score [44], Stereotypic Behavior Rate [45], and Body Condition Score [21]. Models to assess these outcomes were specific to the distribution of the outcome. Foot health consisted of a count score of 0–12, and was tested using Poisson distribution, log link, and deviance scale d. Stereotypy consisted of the average of active scans in January and July, and was tested using Negative Binomial distribution, log link, and deviance scale d. Musculoskeletal health consisted of a 0–3 scale associated with presence of abnormal joints or range of motion, and was tested using multinomial logistic and a cumulative logit link function, with 0 as the reference value. Body condition consisted of assessing “ideal” body condition score 3 compared to elevated body condition scores of 4 or 5, and was tested using a multinomial logistic distribution and a cumulative logit link function. All regression methods utilized an independent correlation matrix. In addition to testing for associations between MDWD and these outcomes at the univariate level, bivariate analyses with age and with species were also conducted.

## Results

### Summary of walking data

**Fig 1:**
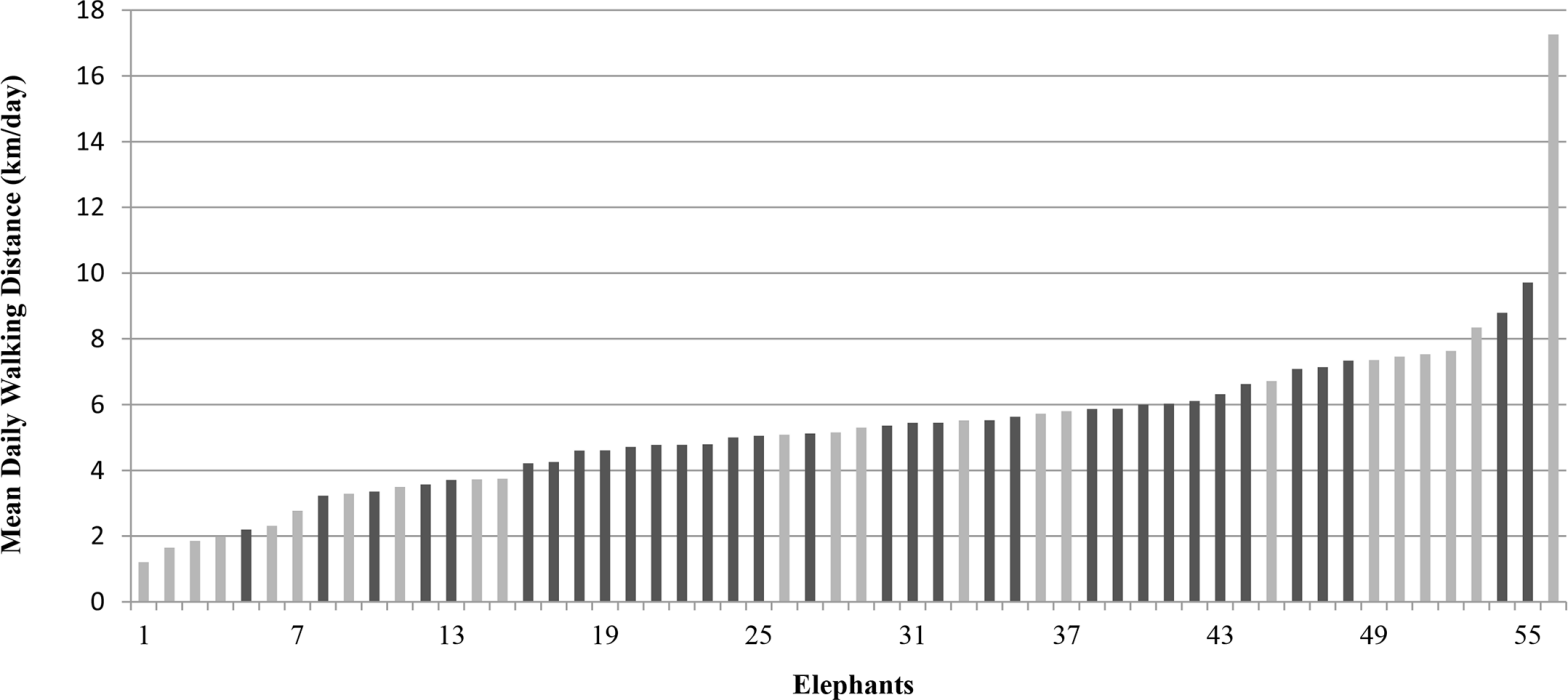
Mean daily walking distance in zoo elephants. Black bars indicate African (n = 33), grey bars indicate Asian (n = 23).

Our final dataset included a total of 259 days of data, collected between May 7, 2012 and November 1, 2012, from 56 elephants at 30 zoos. Five days of data were collected for the majority of elephants (43/56), but for some elephants there were only four days (5/56) or three days (8/56) of data. The 56 elephants included 33 African elephants (58.9%) and 23 Asian elephants (41.1%) (Fig 1). The mean age of the African elephants was 33.2 years (range = 20 to 52) and the mean age of Asian elephants was 40.4 years (range = 16 to 61).

The mean daily walking distance (MDWD) for all elephants was 5.3 km/day. African elephants walked an average of 5.4 km/day and Asian elephants walked an average of 5.3 km/day; there was no significant difference between the species (P = 0.831) (Table 2). There was a large amount of individual variation in MDWD (SD = 2.4 km/day) (Table 2).

**Table 2:** Summary of Mean Daily Walking Distance (km/day) for African and Asian zoo elephants. A t-test used to test for a difference in the means between species was not significant (P = 0.831).

	Combined (n = 56)	African (n = 33)	Asian (n = 23)	P
Mean	5.3	5.4	5.3	0.831
Min.	1.2	2.2	1.2	
Max.	17.3	9.7	17.3	
S.D.	2.4	1.5	3.4	

### Univariate analyses

We evaluated a variety of demographic, housing, social, and management factors for association with MDWD (Table 1). Univariate correlations were observed between MDWD and a number of variables (Table 3). Those variables with correlations where p<0.015 were included in the multi-variable modeling process. MDWD was correlated with 12 housing (space and flooring), eight social variables, and four management variables. MDWD was also correlated with Origin, however, due to the small number of elephants who had been imported from a range country (6/56) it was not possible to interpret these results. The population level descriptive statistics for the variables included in the multi-variable analyses are shown in Table 4.

**Table 3:** Univariate correlations between mean daily walking distance and independent variables.

				Overall			Day ^a^			Night ^a^		
Variables		+/- ^b^	Reference	n	Beta	P-value	n	Beta	P-value	n	Beta	P-value
*Demographics*												
	Age	-		56	-0.084	0.037*						
	Origin		ref ^c^ = Wild	50	0							
		+	Captive	6	1.325	0.049*						
	Species		ref = African	33	0							
		-	Asian	23	-0.144	0.875						
*Space*												
	Total Exhibit Size (500 ft^2^)	+		56	0.003	0.318						
	Space Experience Total (500 ft^2^)	+		54	0.008	0.128^	54	0.005	0.312	54	0.008	0.093^
	Space Experience Indoor (500 ft^2^)	+		54	0.725	0.008*	54	0.390	0.026*	54	0.763	0.005*
	Space Experience Outdoor (500 ft^2^)	+		54	0.008	0.095^	56	0.005	0.131^	54	0.010	0.006*
	Space Experience In/Out Choice (500 ft^2^)		ref = None	19	0		29			21		
		+	Any	37	0.867	0.276	27	1.255	0.103^	35	-0.253	0.797
*Flooring*												
	Percent Time Hard Substrate		ref = None	24	0		28			27		
		-	Any	32	-1.368	0.086^	28	-0.887	0.240	29	-0.599	0.452
	Percent Time Soft Substrate		ref = None	33	0		36			39		
		+	Any	23	1.422	0.107^	20	1.383	0.165	17	1.811	0.097^
*Social*												
	Herd Size	+		54	0.490	0.144^						
	Animal Contact	+		54	0.688	0.086^	54	0.688	0.086^	54	0.626	0.135^
	Social Experience	+		54	0.658	0.208	54	0.410	0.290	54	0.881	0.176
	Social Group Contact			54	0.414	0.060^						
	Percent Time Juveniles (age <7)		ref = None	47	0		47			47		
		+	Any	9	3.137	0.009*	9	3.137	0.009*	9	3.137	0.009*
	Percent Time Housed Separately		ref = None	28	0		38			33		
		-	Any	28	0.564	0.453	18	0.866	0.422	23	-0.589	0.443
*Management*												
	Enrichment Diversity	+		50	3.812	0.071^						
	Enrichment Program	+		50	0.686	0.066^						
	Exercise Diversity	+		50	0.847	0.250						
	Feeding Diversity	+		50	3.086	0.096^					
	Feeding Predictability		ref = 1	13	0							
		+	2	24	0.888	0.293						
		+	3	13	1.864	0.011*						
	Feed Total	+		48	0.122	0.369						
	Alternate Feeding Types	+		56	-0.034	0.662						
	Spread	+		50	-1.586	0.535						
*Other*												
	Temperature	-		56	-0.131	0.059^						
	Recumbence	+		55	-0.187	0.413						

^*P* value <0.15 utilized as threshold significant level for model building

*P value <0.05

^a^ Day and night are defined as the number of hours in a 24 hour period considered daytime or nighttime according to management schedule.

^b^ Hypothesized direction of relationship between mean daily walking distance and variable.

^c^ The reference value (ref =) was the baseline value used when calculating univariate correlations with these binary variables.

**Table 4:** Descriptive statistics for independent variables included in the multi-variable modeling process. The sample size and mean age of elephants used in the correlation is provided.

					Variable				
Variable	Mgmt.	Reference	n	Mean Age	Mean	SD	Min	Max	Median
Age			56	-	36.1	10.4	16	61	34.5
Origin		ref = Wild	50	37.4	-	-	-	-	-
		Captive	6	25.3	-	-	-	-	-
Space Experience Total (500 ft^2^)	Overall		54	36.1	54.5	46.5	12	228.2	33.6
Space Experience Total (500 ft^2^)	Night		54	36.1	46.5	48	2	227.4	29.2
Space Experience Indoor (500 ft^2^)	Overall		54	36.1	1.9	1.5	0	4.7	1.8
Space Experience Indoor (500 ft^2^)	Day		54	36.1	1.9	1.9	0	7.4	1.5
Space Experience Indoor (500 ft^2^)	Night		54	36.1	2	1.6	0	5.2	1.6
Space Experience Outdoor (500 ft^2^)	Overall		54	36.1	71.5	57.8	13	245.3	44.1
Space Experience Outdoor (500 ft^2^)	Day		56	36.1	84	75.9	13	296.9	52.3
Space Experience Outdoor (500 ft^2^)	Night		54	36.1	52.7	59	0	244	36.4
Space Experience In/Out Choice (500 ft^2^)	Day	ref = 0%	29	37.1	-	-	-	-	-
		>0%	27	35.1	44	35.9	6	113	32
Percent Time Hard Substrate	Overall	ref = 0%	24	31.3	-	-	-	-	-
		>0%	32	39.8	12.2	9	1	32	7
Percent Time Soft Substrate	Overall	ref = 0%	33	36.4	-	-	-	-	-
		>0%	23	35.8	19.9	16.4	0	50	18
Percent Time Soft Substrate	Night	ref = 0%	39	36.7	-	-	-	-	-
		>0%	17	34.9	33.6	17.4	15	67	29
Herd Size			54	36.7	3.4	1.7	2	8	3
Animal Contact	Overall, Day		54	36.7	2	1.4	0	6	1
Animal Contact	Night		54	36.7	1.7	1.2	0	5	1
Social Group Contact	Overall		54	36.7	2.8	2.2	1	10	2
Percent Time Juveniles	Overall, Day, Night	ref = 0%	47	37.8	-	-	-	-	-
		>0%	9	27.4	13.2	31	0	100	0
Enrichment Diversity			50	35.4	2.9	0.2	3	3.3	2.9
Enrichment Program			50	35.4	0.3	0.9	-2	2	0.1
Feeding Diversity			50	35.4	1.4	0.3	1	1.8	1.4
Feeding Predictability		ref = 1	13	32.8	-	-	-	-	-
		2	24	39.2	-	-	-	-	-
		3	13	31	-	-	-	-	-

### Multi-variable model

The MDWD multi-variable model (Table 5) includes Beta estimates for Age, Social Group Contact, Space Experience Total Night, Feeding Diversity, and Feeding Schedule. Beta estimates reflect the magnitude of the effect of the independent variables on walking as described below, but it is important to note that this effect is conditional on the effects of the other independent variables in each model. Two feeding variables were included in the multi-variable model. First, elephants with more diverse feeding programs tended to have significantly greater MDWD (P = 0.027). Second, while MDWD did not differ significantly between elephants with predictable feeding schedules and semi-predictable feeding schedules (P = 0.631), elephants with unpredictable feeding schedules walked 1.286 km/day more than elephants with a predictable feeding schedule (P = 0.044). Elephants increased MDWD by 0.422 km/day with every additional social group (P < 0.001), and also decreased their MDWD by 0.023 km for every additional 500 ft^2^ of Space Experience at night (P = 0.009). The model also showed that MDWD decreased by 0.09 km/day for each additional year in age (P = 0.014). Age was included in the models as a confounder of Social Group Contact and Feeding Diversity. Species was included as a confounder of Age.

**Table 5:** Mean daily walking distance multi-variable model (*P < 0.05).

Variable	Beta Estimate	Standard Error	Pr > |Z|	
Intercept	4.01	1.917	0.036	
Age	-0.09	0.037	0.014	*
Species: African	-	-	-	
Species: Asian	1.296	0.644	0.044	*
Social Group Contact	0.422	0.1	<0.001	*
Space Experience Total Night (500 ft^2^)	-0.023	0.009	0.009	*
Feeding Diversity	2.736	1.233	0.027	*
Feeding Predictability: Predictable	-	-	-	
Feeding Predictability: Semi-Predictable	-0.37	0.77	0.631	
Feeding Predictability: Unpredictable	1.286	0.639	0.044	*

### Mean Daily Walking Distance as an Independent Variable

The results of our assessment of the association between MDWD and elephant health and behavioral outcomes are shown in Table 6. We found no significant regression models at the univariate or bivariate (with age or with species) level.

**Table 6:** Mean daily walking distance as input parameter in Generalized Linear Regression Analyses (*P < 0.05).

Outcome	Model	Beta Estimate	RR/OR	Standard Error	95% Confidence Limits		P-Value	Distribution	N
Model 1								Poisson	
Foot Health	MDWD	0.027	1.027	0.085	-0.14	0.194	0.754		51
Model 2								Poisson	
Foot Health	MDWD	0.05	1.06	0.09	-0.12	0.23	0.535		51
Foot Health	Age	0.02	1.02	0.01	-0.01	0.04	0.133		51
Model 3								Poisson	
Foot Health	MDWD	0.04	1.04	0.09	-0.12	0.21	0.610		51
Foot Health	Species	0.32	1.37	0.30	-0.27	0.91	0.293		51
Model 4								Negative Binomial	
Stereotypic Behavior	MDWD	0.235	0.791	0.16	-0.548	0.078	0.141		33
Model 5								Negative Binomial	
Stereotypic Behavior	MDWD	0.01	1.01	0.19	-0.37	0.39	0.951		33
Stereotypic Behavior	Age	0.05	1.05	0.02	0.01	0.08	0.006		33
Model 6								Negative Binomial	
Stereotypic Behavior	MDWD	-0.03	0.97	0.08	-0.20	0.13	0.694		33
Stereotypic Behavior	Species	1.89	6.59	0.51	0.89	2.88	<0.001		33
Model 7								Multinomial Logistic	
Musculoskeletal Health	MDWD	0.341	1.407	0.184	-0.019	0.702	0.063		47
Model 8								Multinomial Logistic	
Musculoskeletal Health	MDWD	0.27	1.30	0.17	-0.07	0.60	0.120		47
Musculoskeletal Health	Age	-0.05	0.95	0.02	-0.09	-0.01	0.018		47
Model 9								Multinomial Logistic	
Musculoskeletal Health	MDWD	0.29	1.34	0.18	-0.07	0.65	0.115		47
Musculoskeletal Health	Species	-0.61	0.54	0.52	-1.62	0.40	0.238		47
Model 10								Multinomial Logistic	
Body Condition	MDWD	-0.144	0.866	0.107	-0.354	0.066	0.179		52
Model 11								Multinomial Logistic	
Body Condition	MDWD	0.11	1.11	0.11	-0.12	0.33	0.350		52
Body Condition	Age	-0.01	0.99	0.03	-0.07	0.05	0.797		52
Model 12								Multinomial Logistic	
Body Condition	MDWD	0.12	1.13	0.11	-0.09	0.33	0.264		52
Body Condition	Species	-0.06	0.94	0.62	-1.28	1.15	0.918		52

## Discussion

There have been several previous single-zoo studies that have assessed zoo elephant walking distances (reviewed in [2]), but different data collection methods and conditions make it difficult to draw comparisons between these studies and our own. In addition, while our methods were certainly informed by previous studies [3,27–28,30], our study is unique in terms of the technology and data processing methods employed and with respect to the breadth of the population studied.

Our results demonstrate that zoo elephants, when housed outdoors for at least 20 hours in a 24 hour period, walk between 1.2 and 17.3 km/day, with individual differences in walking distance associated with demographic, social, housing, and feeding-related variables.

The fact that two feeding-related factors played a significant role in our final model indicates that resource access influences how far zoo elephants walk in a day. The simplest hypothesis regarding this relationship is that when food items are distributed more in space, elephants will to walk more to acquire them. While we were unable to test this relationship explicitly because we could not quantify distances between feeding sites, we did not find support for this hypothesis when we tested the variable Spread, which measured the frequency with which food was distributed across the exhibit rather than concentrated in specific areas. By contrast, the features of feeding programs that were found to be positively associated with walking were the degree to which the timing of feedings was unpredictable and the diversity in the methods used to present food. One explanation for this combination of results is that that more dynamic feeding regimens lead to an increase in exploratory behavior, which elephants express via locomotion. Shepherdson et al. [12] found that leopard cats that were switched from a temporally predictable feeding schedule to an unpredictable feeding schedule with food items hidden throughout the enclosure increased their exploratory behavior. Higher feeding diversity scores, which indicate use of browse, food puzzles, and hanging food items in addition to trough or floor feeding, were also associated with increased locomotion, possibly because diversity in feeding opportunities promotes physical exploration of the exhibit. However, it is also possible that the increase in walking that we found related to feeding predictability and diversity is a response to increased arousal or frustration caused by the dynamic feeding regimens.

There was one social variable in the multi-variable model as well. Social Group Contact is a variable that quantifies the common management practice of dividing the herd into various sub-groups, with individual elephants spending time in one or more of these social configurations [36]. In our study population, the elephants spent time in between 1 and 10 different social groups. As an elephant’s Social Group Contact score went up, walking distances increased. A larger number of social groups could better replicate a fission-fusion society in which core social groups temporarily divide and reunite over the course of hours or days [46–50]; increased walking could be the result of elephants walking more to interact socially as social groups change and could be a form of social exploration. However, in a related study, Brown et al. [51] found that Social Group Contact was positively associated with hyperprolactinemia in female elephants, which the authors interpreted as a physiological response to social stress caused by dynamic social management. Future studies could delve more deeply into the correlation we found by recording both the quality of social interactions and the amount of walking associated with different social management regimens.

Perhaps most interesting is the fact that none of our measures of space either at the zoo level (Exhibit) or the individual elephant level (Space Experience) were positively associated with distance walked in the multi-variable model. In fact, we found increased Space Experience at night was negatively correlated with distance walked. However, the magnitude of this effect was quite small. This finding challenges the idea that elephants will voluntarily walk greater distances if provided with more room to do so, and in combination with our feeding related results, indicates that exhibit space may be important to walking distances mainly to the extent that it supports the application of diverse and dynamic feeding programs. In considering this lack of effect, is important to note that the range of exhibit sizes in our study population may not have been sufficient to demonstrate the impact that extensive areas can have on walking distances. Studies of wild elephants in non-extreme environmental conditions indicate that average daily walking distances range from 5–10 km/day when there is extensive space availability. Since our measured walking distances are at the lower end of this range, it is possible that elephants would walk more if housed in exhibits larger than those currently represented in the North American zoos participating in this study.

However, setting wild walking distances as a benchmark for managed elephant programs or operating under the assumption that walking distances should be increased as a goal of zoo elephant management is only appropriate if it can be demonstrated that walking is important to elephant welfare. Because our study was conducted as part of the Using Science to Understand Zoo Elephant Welfare Project [25], we were able to test for associations between walking distances and several welfare indicators including foot health, joint health, body condition and stereotypic behavior.

Stereotypic behavior is considered a strong indicator of compromised welfare and is used extensively to evaluate the welfare of animals in a variety of managed settings [52–53]. Locomotor stereotypies such as pacing are a concern for many zoo-housed species [54–55] and long bouts of pacing by elephants could have inflated our measurements of distance walked. Since we did not combine our GPS data collection with direct behavioral observations, we cannot state conclusively that the elephants in our study did not perform locomotor stereotypy. However, in a related study that included 33 of our study elephants, Greco et al. [45] demonstrated that locomotor stereotypy is infrequently performed by North American zoo elephants and that the vast majority (over 90%) of the stereotypy elephants perform is non-ambulatory and consists of rocking and swaying. These most frequently performed forms of stereotypy should have limited effect on distance measurements collected with GPS anklets [28]. Additionally, the development of stereotypic behaviors in animals is associated with environments that inhibit the performance of highly motivated behaviors [53], and as such, if elephants are motivated to walk and cannot due to environmental restrictions, stereotypic behaviors could develop as a result. However our analysis of walking as an independent variable for stereotypic behavior showed no correlation. As such, we found no evidence to support the conclusion that elephants who walk shorter distances are more likely to perform higher rates of stereotypic behavior.

We also tested the relationship between walking distances and foot and musculoskeletal health because foot and joint conditions are the most commonly reported health issues affecting African and Asian elephants in zoos [20, 56] and it has been suggested that limited walking is a risk factor for these conditions [57]. We found no correlations between walking distance and either foot or musculoskeletal health. Similarly, Miller et al. determined that age and time spent on hard flooring substrates, not distances walked, were the primary predictors of both foot and joint problems in a related study [44]. It must be noted that while the foot and musculoskeletal health data and our walking distance data were collected on the same elephants during the same calendar year, they were not collected at exactly the same time. Although foot problems have been found to be persistent over time for a large proportion of this population [43], it is possible that measures of foot and musculoskeletal health taken coincident with walking distance measurements could show associations that we did not find.

Finally, we tested for an association between mean daily distances walked by elephants and body condition scores. These scores were assessed on a scale of 1–5 (see [21]) during the same calendar year as walking distances were measured. Body condition is a measure of fat deposition and is considered a welfare indicator for elephants because of plausible associations with conditions such as cardiovascular disease, arthritis and foot problems, and ovarian cycle abnormalities [20,57–64], and greater walking distances have been predicted to be associated with increased likelihood of ideal body condition [65]. However, our assessment showed that elephants who walked more were not more likely to have ideal body condition. This lack of association could potentially be explained by the importance of the feeding program variable, Feeding Diversity, to both walking distances and risk of elevated body condition. Our models show that greater walking distances are associated with higher Feeding Diversity scores, but interestingly, Morfeld et al. [21] report that elephants who experience more diverse feeding programs are at higher risk of being overweight. If Feeding Diversity scores are a proxy for the amount of food an elephant eats (as hypothesized by Morfeld et al.), then perhaps the energetic expenditure of walking is being offset by caloric intake and therefore the predicted association between walking and ideal body condition was not found.

Overall, we found that the distances zoo elephants walk are influenced most significantly by feeding-related factors, and these associations suggest that walking in zoo elephants may be an expression of exploratory behavior. However more research is necessary to determine explicitly how differences in opportunity to explore via walking may impact elephant welfare, as we found no associations between distances walked and the behavioral or health outcomes we tested.
